# Low awareness of viral causes of Cancer among United States adults by smoking status: findings from health information national trends survey (2024)

**DOI:** 10.1016/j.pmedr.2026.103466

**Published:** 2026-04-04

**Authors:** Wenxue Lin, Shengyang Wang, Lin Ge

**Affiliations:** Department of Epidemiology and Biostatistics, School of Public Health, Indiana University Bloomington, Bloomington, IN 47405, USA

**Keywords:** Risk factors, Hepatitis B virus, Hepatitis C virus, Smokers, Health surveys

## Abstract

**Objective:**

Viral infections such as hepatitis B virus (HBV) and hepatitis C virus (HCV) are major risk factors of liver cancer, and smoking may modify this risk. This study aimed to quantitively assess differences in awareness of HBV and HCV as cancer risk factors by smoking status among U.S. adults.

**Methods:**

Data were obtained from the 2024 Health Information National Trends Survey (HINTS 7) with *N* = 5860. Awareness was categorized as “yes” versus “no,” “not sure,” or “never heard.” Differences in awareness of HBV or HCV as cancer risk factors by smoking status were examined univariately using survey-weighted chi-square tests, as well as using weighted logistic regression models adjusting for demographic characteristics.

**Results:**

Awareness of HBV as a cancer risk factor was low and did not differ significantly by smoking status (current smokers, 17.2%; former smokers, 18.7%; non-smokers, 18.0%; *p* = 0.88). Similarly, HCV awareness was 16.7%, 16.9%, and 16.7%, respectively. In multivariable analyses, smoking was not significantly associated with awareness of HBV (*p* = 0.13) or HCV (*p* = 0.36).

**Conclusions:**

Awareness of viral hepatitis as a cancer risk factor remains low regardless of smoking status, underscoring the need for improved public health education, vaccination, and screening.

## 1 Introduction

Hepatitis B virus (HBV) and hepatitis C virus (HCV) are major risk factors for liver cancer and contribute substantially to the global cancer burden (World Health Organization, 2017). Evidence suggests that smoking could modify this risk, even though it is not a primary etiologic cause of liver cancer, with smokers experiencing a higher risk (Shadi et al., 2024). Smoking status may influence awareness of viral hepatitis–related liver cancer risk. Smokers may have greater exposure to cancer-related massages through clinical encounters or public health campaigns. Conversely, smokers often face barriers to healthcare access (Teferra et al., 2023), which may further limit opportunities for screening, prevention, and treatment of viral hepatitis. Despite the availability of effective vaccination and antiviral therapies, awareness of the link between viral infections and cancer remains limited in many populations, regardless of smoking status (Colvin and Mitchell, 2010). Examining awareness by smoking status thus provides insight into whether elevated epidemiologic risk and potential risk stratification translate into differences in knowledge. Therefore, this study aimed to quantitively assess differences in awareness of HBV and HCV as cancer risk factors by smoking status among U.S. adults.

## 2 Methods

### 2.1 Study design and population

Data were obtained from the 2024 Health Information National Trends Survey (HINTS 7), a nationally representative mail survey of non-institutionalized U.S. adults aged 18 years and older, conducted by the National Cancer Institute and administered from March through September 2024 (Westat, 2025). HINTS 7 (2024) collects information on health behaviors, cancer-related knowledge, and information-seeking patterns using a stratified, two-stage sampling design (Westat, 2025). The survey instruments undergo extensive development procedures, including expert review, cognitive testing, and field testing to ensure content validity and interpretability (Finney Rutten et al., 2020). The public use dataset includes replicate weights to account for complex survey design, enabling accurate population-level estimates (Westat, 2025).

### 2.2 Measures and statistical analysis

All respondents with complete data on smoking status and awareness of HBV and HCV as cancer risk factors were included in the analysis. Awareness was assessed using the questions: “Do you think the Hepatitis B virus (also known as Hep B or HBV) can cause cancer?” and “Do you think the Hepatitis C virus (also known as Hep C or HCV) can cause cancer?” Awareness of HBV and HCV was dichotomized as “yes” versus “no/not sure/never heard.” Smoking status was categorized into three groups: current smoker, former smoker, and non-smoker (Lin and Muscat, 2021).

Weighted descriptive statistics were reported to characterize the study population, including demographic characteristics (sex, age group, race/ethnicity, education and income). Due to small sample sizes in several racial/ethnic subgroups, race/ethnicity was categorized into broader groups in the main analysis to ensure stable estimates and sufficient statistical power; the “Non-Hispanic (NH) Other” category includes American Indian or Alaska Native, Asian, Pacific Islander, and other less-represented groups. Survey-weighted chi-square tests were conducted to examine differences in awareness of HBV or HCV as cancer risk factors, as well as differences in demographic characteristics by smoking status among U.S. adults. Furthermore, weighted logistic regression models were conducted to estimate odds ratios (OR) and 95% confidence intervals (Cl) for awareness of HBV or HCV as cancer risk factor by smoking status, adjusting for all demographic characteristics.

All analyses were conducted using R (version 4.3.3). The survey package was used to account for the complex sampling design and replicate weights provided in HINTS 7 (Westat, 2025). Variance estimates, standard errors, and confidence intervals were computed using the replicate-weight method to ensure design-consistent inference. Of the 7278 respondents, approximate one-fifth were excluded due to missing values, resulting in a final analytic sample of *N* = 5860.

The HINTS has been approved through expedited review from the Westat Institutional Review Board and is exempt from the United States National Institutes of Health Office of Human Subjects Research Protections. The present analysis used publicly available, de-identified data from National Cancer Institute website (https://hints.cancer.gov/). Therefore, additional institutional review board approval and informed consent were not required.

## 3 Results

Awareness of HBV and HCV as causes of cancer was low and did not differ significantly across all smoking groups. As shown in Table 1 and Supplementary Fig. 1., only 17.2%, 18.7%, and 18.0% of current, former, and non-smokers were aware (answered “Yes”) that HBV infection contributes to cancer (*p* = 0.88). Similarly, 16.7%, 16.9%, and 16.7% of participants of current, former, and non-smokers recognized the association between HCV and cancer (*p* = 0.99).

**Table 1:** Demographic Characteristics of U.S. Adult Participants Stratified by Smoking Status in the Health Information National Trends Survey (HINTS 7), 2024.

	Current Smoker *N* = 600	Former Smoker *N* = 1491	Non-Smoker *N* = 3769	*p*-value⁎
	n (%)	n (%)	n (%)	
**Age group**				**<0.01**
18–34	97 (20.9)	107 (11.1)	795 (31.5)	
35–49	156 (33.5)	250 (23.8)	866 (27.5)	
50–64	193 (30.7)	384 (31.5)	974 (25.0)	
65–74	112 (10.7)	443 (20.1)	679 (9.7)	
75+	42 (4.2)	307 (13.4)	455 (6.2)	
**Sex**				**<0.01**
Male	263 (53.4)	712 (57.5)	1389 (48.6)	
Female	337 (46.6)	779 (42.5)	2380 (51.4)	
**Race/ethnicity**#				**<0.01**
Hispanic	102 (12.6)	187 (10.1)	890 (20.3)	
NH White	323 (63.8)	1043 (76.9)	1897 (55.4)	
NH Black	107 (11.4)	168 (6.7)	605 (12.4)	
NH Other	68 (12.2)	93 (6.3)	377 (11.9)	
**Education**				**<0.01**
less than high school	85 (13.1)	72 (4.7)	181 (4.3)	
high school graduate	138 (29.1)	249 (18.7)	529 (19.0)	
some college	224 (43.5)	511 (46.7)	951 (35.0)	
college graduate or more	153 (14.3)	659 (29.9)	2108 (41.7)	
**Income**				**<0.01**
less than $20,000	196 (35.4)	194 (8.4)	549 (12.2)	
$20,000 to $35,000	98 (13.6)	198 (11.3)	413 (10.6)	
35,000 to $50,000	80 (11.2)	217 (13.8)	418 (10.4)	
50,000 to $75,000	102 (15.7)	268 (17.4)	609 (15.7)	
75,000 to $100,000	47 (7.5)	195 (15.7)	492 (12.3)	
100,000 or greater	77 (16.5)	419 (33.4)	1288 (38.6)	
**HBV causes cancer**				0.88
Yes	95 (17.2)	270 (18.7)	738 (18.0)	
No or don't know	505 (82.8)	1221 (81.3)	3031 (82.0)	
**HCV causes cancer**				0.99
Yes	94 (16.7)	254 (16.9)	713 (16.7)	
No or don't know	506 (83.3)	1237 (83.1)	3056 (83.3)	

⁎ Note: p-value from survey-weighted chi-square tests.

# NH: non-Hispanic HINTS: Health Information National Trends Survey.

Significant differences in demographic characteristics were observed across smoking groups (all *p* < 0.01; Table 1). Current smokers were more likely to be middle-aged, male, and have lower education and income, whereas non-smokers were more likely to be younger, female, and have higher socioeconomic status. Former smokers tended to be older and had intermediate socioeconomic characteristics.

In multivariable analyses (Table 2), no significant differences in awareness were observed by smoking status. Compared with non-smokers, adjusted odds ratios (ORs) for HBV awareness as cancer risk factor were 1.19 (95% CI: 0.77–1.83) for current smokers and 1.31 (95% CI: 1.01–1.72) for former smokers (*p* = 0.13). Likewise, adjusted OR for HCV awareness as cancer risk factor were 1.18 (95% CI: 0.80–1.75) and 1.22 (95% CI: 0.89–1.66), respectively (*p* = 0.36).

**Table 2:** Adjusted Odds Ratios (ORs) for Awareness of Hepatitis B Virus (HBV) or Hepatitis C Virus (HCV) Infection among U.S. Adult Participants, HINTS 7 (2024).

	HBV	HCV
	Adjusted OR (95% CI)	Adjusted OR (95% CI)
**Smoker status**⁎		
Non-smoker	1.00	1.00
Former smokers	1.31 (1.01, 1.72)	1.22 (0.89, 1.66)
Current smokers	1.19 (0.77, 1.83)	1.18 (0.80, 1.75)

**Sex**		
Female	1.00	1.00
Male	0.87 (0.67, 1.13)	0.89 (0.69, 1.17)

**Age group**		
18–34	1.00	1.00
35–49	1.12 (0.72, 1.73)	1.30 (0.79, 2.16)
50–64	0.86 (0.60, 1.23)	0.94 (0.66, 1.34)
65–74	0.76 (0.50, 1.17)	0.83 (0.53, 1.32)
75+	0.45 (0.29, 0.69)	0.45 (0.25, 0.81)

**Race/ethnicity**		
Hispanic	1.00	1.00
NH White	1.10 (0.76, 1.59)	1.24 (0.87, 1.75)
NH Black	1.27 (0.86, 1.89)	1.29 (0.79, 2.11)
NH Other	1.53 (0.98, 2.37)	1.38 (0.82, 2.34)

**Education**		
less than high school	1.00	1.00
high school graduate	1.09 (0.44, 2.71)	0.81 (0.31, 2.14)
some college	1.21 (0.57, 2.55)	1.07 (0.50, 2.28)
college graduate or more	2.29 (1.04, 5.01)	1.87 (0.88, 3.95)

**Income**		
less than $20,000	1.00	1.00
$20,000 to $35,000	0.72 (0.39, 1.33)	0.76 (0.34, 1.69)
35,000 to $50,000	0.69 (0.34, 1.37)	0.59 (0.28, 1.24)
50,000 to $75,000	0.82 (0.43, 1.59)	0.67 (0.33, 1.39)
75,000 to $100,000	0.90 (0.48, 1.68)	0.71 (0.37, 1.35)
$100,000 or greater	1.01 (0.54, 1.86)	0.96 (0.48, 1.93)

⁎ Note: Wald-type p-values for the ORs of smoking status were 0.13 for HBV and 0.36 for HCV. As the primary aim of this study was to examine differences in awareness of HBV and HCV as cancer risk factors by smoking status, emphasis was placed on the statistical significance (*p*-values) rather than the magnitude of the ORs.

In addition, older age (≥75 years) was associated with significantly lower awareness of both HBV and HCV as cancer risk factors, while higher educational attainment was associated with greater awareness, particularly for HBV. No consistent associations were observed for sex, race/ethnicity, or income.

## 4 Discussion

Chronic HBV and HCV infections are well-established causes of hepatocellular carcinoma and contribute significantly to the cancer burden in the United States. Globally, the burden of viral hepatitis remains substantial. There were an estimated 1.2 million new infections with the hepatitis B virus and 1 million new infections with the hepatitis C virus (World Health Organization, 2024). The prevalence of chronic infections is even more significant, affecting approximately 254 million people with chronic hepatitis B and nearly 50 million with chronic hepatitis C (World Health Organization, 2024).

This study addresses an important public health issue by examining awareness of HBV and HCV as cancer risk factors in the context of their high prevalence and substantial contribution to liver cancer. A key strength of this work is the use of complementary quantitative approaches to assess awareness and its variation by smoking status. Descriptive analyses provided population-level estimates and univariate comparisons of awareness of HBV and HCV as cancer risk factors across smoking groups, while weighted logistic regression models evaluated these differences after adjusting for demographic characteristics. This combined analytical framework enhances the robustness of the findings and allows for a more nuanced understanding of awareness patterns across population subgroups.

Our study's results align with other evidence showing low awareness of hepatitis infections among U.S. adults. For instance, one population-based study found that fewer than half of U.S. adults with HBV or HCV infection were aware of their status; awareness of HBV infection was approximately 32% and for HCV about 49% (Zhou and Terrault, 2020). Another analysis of HCV awareness reported that the weighted awareness of infection remained about 60%, indicating that more than 800,000 U.S. adults may be unaware of their HCV infection (Gnanapandithan and Ghali, 2023). Taken together, these findings suggest a dual problem: lack of awareness both of infection status and of the role of these viral infections in carcinogenesis.

Importantly, we observed a strong educational gradient in awareness. Individuals with higher educational attainment had substantially greater odds of recognizing the link between viral hepatitis and liver cancer. This gradient likely reflects structural disparities in health literacy, access to health information, and engagement with preventive care. Higher education is consistently associated with greater exposure to health messaging and improved ability to interpret medical information. Our findings suggest that awareness of viral hepatitis–related cancer risk is socially patterned, underscoring the need for targeted communication strategies aimed at populations with lower educational attainment.

In addition, the analyses in this study identified no significant differences in awareness of HBV or HCV by smoking status, indicating a strong need for broadly targeted educational efforts, especially for smokers. Certain sociodemographic groups, such as low-income individuals, those with limited educational attainment, and people with restricted healthcare access, are more likely to smoke and simultaneously face barriers to screening and vaccination. Smoking may act synergistically with chronic viral hepatitis to accelerate liver damage and carcinogenesis. Multiple studies have suggested that the carcinogenic toxicants in tobacco smoke may promote inflammation and fibrosis in hepatitis-infected livers, thereby exacerbating cancer risk (Marti-Aguado et al., 2022; Premkumar and Anand, 2021).

From a public health perspective, raising awareness of the viral causes of liver cancer is critical for prevention. Improved public awareness can increase acceptance of HBV vaccination, enhance HCV screening, and encourage early detection of liver disease. Primary care visits and tobacco cessation counseling offer key opportunities for providers to engage in patient education about HBV vaccination and HCV screening. Strengthening provider communication may bridge the gap between awareness and action, ultimately reducing the burden of liver cancer associated with viral hepatitis and smoking.

There are several limitations to our study. We employed a complete-case analysis and assumed that data were missing completely at random. If missingness was associated with health literacy, smoking status, or awareness, this approach may have introduced selection bias. In addition, awareness was dichotomized, combining “no,” “not sure,” and “never heard.” These categories may reflect distinct levels of knowledge, and such grouping may have introduced non-differential misclassification, potentially attenuating associations, particularly among older adults or medically complex individuals who may experience recall difficulties. (Castro and Plus, 2022).

Furthermore, liver cancer burden due to HBV and HCV varies across racial and ethnic subgroups, particularly among Asian and Pacific Islander populations (Pinheiro et al., 2025). Future studies pooling multiple years of HINTS data could allow more granular subgroup analyses of awareness patterns and enhance statistical power. Future research could also examine behavioral or quality-of-life outcomes to better contextualize awareness as a potential intermediate endpoint (Duan et al., 2026). Moreover, exploring potential interaction effects between smoking status and other sociodemographic factors may provide further insight into subgroup differences in awareness.

## 5 Conclusion

Awareness of HBV and HCV as cancer risk factors remains concerningly low among U.S. adults and does not differ significantly by smoking status. These findings suggest that elevated epidemiologic risk among smokers is not accompanied by greater awareness, highlighting missed opportunities for targeted health communication.

From a public health perspective, these findings underscore the urgent need for enhanced public health education that integrates viral hepatitis with cancer prevention efforts, particularly among smokers. Interventions should prioritize improving health literacy, particularly among populations with lower educational attainment, and leverage clinical settings, such as primary care and smoking cessation programs, to promote HBV vaccination and HCV screening.

Strengthening awareness may serve as a critical first step toward increasing uptake of preventive services and reducing the burden of liver cancer. Future research should further explore subgroup disparities and the broader behavioral implications of awareness to better inform targeted prevention strategies.

## CRediT authorship contribution statement

**Wenxue Lin:** Writing – review & editing, Writing – original draft, Methodology, Formal analysis, Conceptualization. **Shengyang Wang:** Writing – review & editing, Visualization, Software, Methodology, Formal analysis. **Lin Ge:** Writing – review & editing, Methodology, Investigation, Formal analysis.

## Funding

Publication of this article was supported by faculty start-up funds from School of Public Health-Bloomington, Indiana University.

## Declaration of competing interest

The authors declare that they have no known competing financial interests or personal relationships that could have appeared to influence the work reported in this paper.

## Appendix A Supplementary data

Supplementary material Supplementary Figure 1: Awareness of Hepatitis B Virus (HBV) and Hepatitis C Virus (HCV) Infection by Smoking Status among U.S. Adult Participants in HINTS 7, 2024.
