# Supplementary material for: Low awareness of viral causes of Cancer among United States adults by smoking status: findings from health information national trends survey (2024)
Source: Prev Med Rep. 2026 Apr 4;65:103466. doi: 10.1016/j.pmedr.2026.103466 (PMC13090952; doi:10.1016/j.pmedr.2026.103466)
Supplement: Supplementary material — Supplementary Figure 1: Awareness of Hepatitis B Virus (HBV) and Hepatitis C Virus (HCV) Infection by Smoking Status among U.S. Adult Participants in HINTS 7, 2024. [file mmc1.docx]

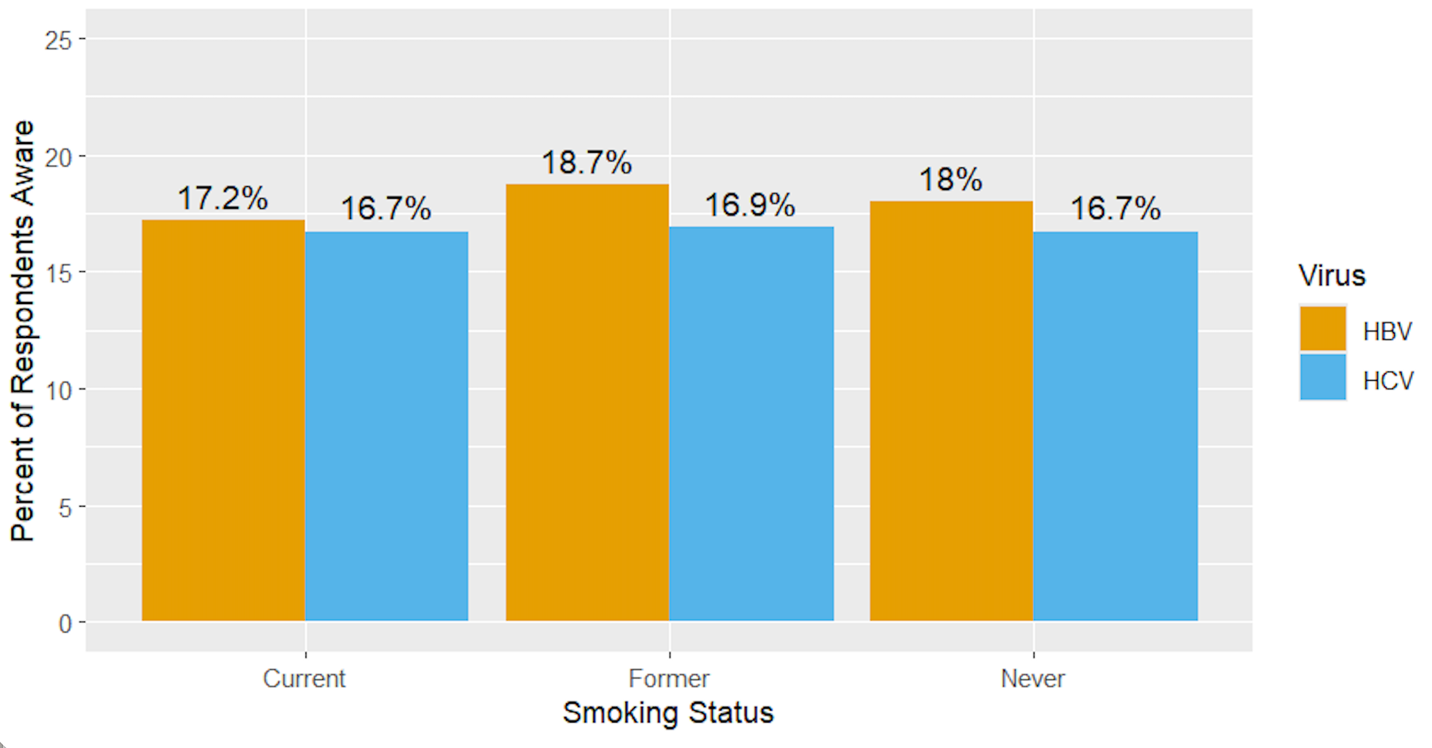


Figure 1 Awareness of Hepatitis B Virus (HBV) and Hepatitis C Virus (HCV) Infection by Smoking Status among U.S. Adult Participants in HINTS 7, 2024
